# Authors’ response: Excess all-cause mortality during second wave of COVID-19 – the Polish perspective

**DOI:** 10.2807/1560-7917.ES.2021.26.7.2100191

**Published:** 2021-02-18

**Authors:** Lasse S Vestergaard, Sarah K Nørgaard, Jens Nielsen, Tyra G Krause, Kåre Mølbak

**Affiliations:** 1 EuroMOMO hub, Statens Serum Institut, Copenhagen, Denmark

**Keywords:** all-cause mortality, COVID-19 pandemic, Europe, EuroMOMO

**To the editor:** We sincerely welcome and appreciate the comments and contributions from Grabowski et al. concerning our recent report on the excess morality burden in Europe during the ongoing second wave of the coronavirus disease (COVID-19) pandemic [1]. Their sharing of excess mortality data and perspectives from Poland and other European countries that are not yet part of the European monitoring of excess mortality for public health action (EuroMOMO) network (www.euromomo.eu) certainly adds important information and some critical dimensions in revealing the full impact of the COVID-19 pandemic as it has evolved since early 2020. The all-cause mortality figures from Poland show how the COVID-19 pandemic has led to a difficult situation in the country, leaving no question about the dramatic excess death toll and how it has burdened the health system and people [2].

These additional data allow us to better comprehend the full scale of the COVID-19-related mortality across Europe, and also emphasise the significant combined direct and indirect burden posed by the pandemic. Furthermore, those indirect effects of the pandemic highlight the important added value of monitoring all-cause mortality, in addition to COVID-19-specific mortality. A considerable number of people infected with severe acute respiratory coronavirus 2 (SARS-CoV-2) will not be tested and may thus result in COVID-19-related deaths not being detected and reported; however, the additional burden on constrained national health services adds to the mortality figures. The all-cause mortality data can help us estimate, in real time, the full impact of infectious disease threats, informing here and now any necessary national public health action as well as informing the general public [3]. Indeed, the mortality reports from EuroMOMO have been read widely, telling where and whom the pandemic has affected as it has evolved, week by week. In addition, EuroMOMO’s weekly outputs have been used by international health agencies such as the European Centre for Disease Prevention and Control (ECDC) and the World Health Organization (WHO) for their rapid risk assessments and other updates and guidance to countries during the pandemic [4,5]. Both organisations also actively encourage and support countries in implementing the EuroMOMO weekly mortality monitoring system as part of their national pandemic preparedness.

The EuroMOMO network currently includes 27 participating countries or subnational areas, covering a population of around 300 million people in Europe. So far, three additional countries have joined the network during the pandemic and other countries have expressed their interest, because of the urgent need for timely monitoring of COVID-19-related mortality. Certainly, we warmly welcome new countries in the network. From our hub in Copenhagen we have regularly conducted workshops for new member countries and offered technical support to set up the system at operational level in these countries. The weekly application of the EuroMOMO algorithm immediately provides national authorities with a simple and easy-to-use system for national purposes, while at the same time contributing essential information to understand the mortality from COVID-19 and other infectious disease threats, including seasonal influenza, at the wider European level. Countries interested to join EuroMOMO are most welcome to make contact and we look forward to working with them.
